# The UP-TECH project, an intervention to support caregivers of Alzheimer’s disease patients in Italy: study protocol for a randomized controlled trial

**DOI:** 10.1186/1745-6215-14-155

**Published:** 2013-05-28

**Authors:** Carlos Chiatti, Filippo Masera, Joseph M Rimland, Antonio Cherubini, Osvaldo Scarpino, Liana Spazzafumo, Fabrizia Lattanzio

**Affiliations:** 1 Italian National Research Center on Aging (INRCA), Ancona, Italy

**Keywords:** Alzheimer’s disease, Technology, Caregiver burden, Quality of life, Integrated care, RCT, Italy

## Abstract

**Background:**

The epidemic of Alzheimer's disease (AD) represents a significant challenge for the health care and social service systems of many developed countries. AD affects both patients and family caregivers, on whom the main burden of care falls, putting them at higher risk of stress, anxiety, mortality and lower quality of life. Evidence remains controversial concerning the effectiveness of providing support to caregivers of AD patients, through case management, counseling, training, technological devices and the integration of existing care services. The main objectives of the UP-TECH project are: 1) to reduce the care burden of family caregivers of AD patients; and 2) to maintain AD patients at home.

**Methods/design:**

A total of 450 dyads comprising AD patients and their caregivers in five health districts of the Marche region, Italy, will be randomized into three study arms. Participants in the first study arm will receive comprehensive care and support from a case manager (an *ad hoc* trained social worker) (UP group). Subjects in the second study arm will be similarly supported by a case manager, but in addition will receive a technological toolkit (UP-TECH group). Participants in the control arm will only receive brochures regarding available services. All subjects will be visited at home by a trained nurse who will assess them using a standardized questionnaire at enrollment (M0), 6 months (M6) and 12 months (M12). Follow-up telephone interviews are scheduled at 24 months (M24). The primary outcomes are: 1) caregiver burden, measured using the Caregiver Burden Inventory (CBI); and 2) the actual number of days spent at home during the study period, defined as the number of days free from institutionalizations, hospitalizations and stays in an observation unit of an emergency room.

**Discussion:**

The UP-TECH project protocol integrates previous evidence on the effectiveness of strategies in dementia care, that is, the use of case management, new technologies, nurse home visits and efforts toward the integration of existing services in an ambitious holistic design. The analysis of different interventions is expected to provide sound evidence of the effectiveness and cost of programs supporting AD patients in the community.

**Trial registration:**

ClinicalTrials.gov: http://NCT01700556

## Background

The *World Alzheimer Report* estimated that there were 35.6 million people living with dementia worldwide in 2010, and according to forecasts this figure will reach 65.7 million by 2030 and 115.4 million by 2050 [1]. The Italian National Institute of Statistics (Istat) estimated that there were approximately 250,000 people suffering from Alzheimer’s disease (AD) and similar dementias in Italy in 2005 [2]. However, since AD and various forms of age-related cognitive deterioration have complex diagnoses, their prevalence is likely to be underestimated [3,4].

AD causes progressive cognitive and functional decline [5], it can have a significant impact on care costs, and is the major cause of nursing home admission [6]. To accurately estimate the societal impact of AD, one needs to consider that patients' families are also affected, since the burden of care in many countries, including Italy, mainly falls on them. AD is known as a ‘family illness’, because family caregivers of AD patients often represent hidden secondary patients [7]. They frequently experience high levels of stress, associated with a higher risk of developing mood disorders, depression, insomnia and a lower quality of life [8]. Anxiety and stress also increase their likelihood of developing physical problems, such as headache, back pain and excess weight, and exposes them to a higher rate of mortality compared to their non-caregiver counterparts [9,10].

Despite controversies [11], promising intervention studies have shown that specific interventions aimed at supporting caregivers of AD patients can lead to significant improvements in their physical and mental health, by reducing caregiver burden and stress [12]. For instance, caregivers receiving specific counseling sessions provided care for longer and delayed the institutionalization of their relatives with AD [13-17]. Counseling of family members delayed admission of the patient to a long-stay facility for an average of 18 months [13]. An experimental program of intensive training, followed by 10 days of follow-up contacts, delayed the institutionalization and reduced the level of caregiver stress [13,18]. In the USA, telephone-based psychosocial support was effective at lowering caregiver stress [19], suggesting that such interventions can provide an interesting low-cost solution to support caregivers.

In this context, new technologies applied to the home hold great potential to improve the quality of life of AD patients and their caregivers. Despite the absence of definitive evidence [20], pilot studies revealed that home adaptations designed to assist older people in fulfilling their daily needs in the home are particularly important for users' quality of life and well-being [21]. Several studies assessing the effectiveness of assistive technology in AD have been undertaken; however, most of these interventions are in a prototype/testing phase and fail to be scaled-up and systematically implemented in daily practice. A few of these include: talking lights, the Home Assurance System project [22], the ROSETTA project [23] and the ENABLE project [24]. The latter project involved the testing of an integrated system of technologies in the home, including automatic night lights, locators for lost objects, computerized calendars, programmable telephones, gas sensors, touch screen pads for music and a voice prompter when medicines should be taken.

One of the largest intervention studies undertaken in the field is the Resources for Enhancing Alzheimer's Caregiver Health (REACH) project, which was launched in the USA in 1995 [25]. Sponsored by the National Institute on Aging and the National Institute of Nursing Research, the main objective was to develop a psychosocial intervention to improve the living conditions of caregivers of AD patients. The REACH project implemented a multi-component intervention, including: 1) individual support of caregivers; 2) group support and family therapy; 3) training of caregivers; 4) interventions to adapt to the home; and 5) the use of technology support systems (for example a telephone-linked computer system and a computer telephone integration system). Evidence suggests that the program was effective at both improving caregivers' quality of life and in delaying the institutionalization of AD patients [8,26], and even today this multi-site intervention represents a major testbed for evidence-based evaluation of practices in the field of dementia care.

An important issue to consider when implementing programs to improve care pathways for AD patients is that the care of these patients is an extremely complex process. Public welfare professionals must take into account multiple care needs (for example health, social and legal), without neglecting the importance of the caregiver and their needs as well. The multidimensionality of this problem not only calls for new services, but also for a greater coordination and integration of existing community health care and social services, within the public, non-profit and private sectors. This was the assumption underlying several projects integrating health care and social services, thus reducing cost, and eliminating waste and inefficiency [27-30]; however, none of these specifically addressed AD patients and their caregivers. Among the tools used in these studies to foster integration of care are case management strategies, staff training and the use of new information technology (IT) systems.

The Italian welfare system has traditionally relied on a family-based approach to older people care. Here, the level of formal care provision has always been marginal when compared to other Western welfare systems, such as those of the UK, Germany and Scandinavian countries [31]. However, stimulated by the increasing dimension of AD and its consequences on families, the Italian Ministry of Social Welfare decided to invest in the innovation of care services in this field. A call for proposals was issued in 2010 addressing public service providers, with the aim of developing innovative interventions, and improving the effectiveness of care for AD patients and their caregivers in the Italian regions.

Responding to this call and building on the knowledge retrieved in the literature, the Italian National Research Centre on Aging (INRCA) in partnership with the Government of the Marche region, developed the proposal of the UP-TECH intervention study, which was successfully evaluated and funded with a grant of approximately 1 million euros. The ambitious idea underlying this project is to create a multi-component intervention program at regional level, which could engage professionals working in both the social and health care services, in a large-scale organizational change to restructure the processes of AD care. For these reasons, a study has been designed following the trial methodology and adapting to the Marche region the most promising evidence-based interventions in this area.

The main project objectives of the UP-TECH study are: 1) to reduce the care burden of family caregivers of AD patients; and 2) to maintain AD patients at home.

Secondary objectives of the project are: 1) to ensure continuity of care and the integration of care pathways; 2) to create an information management system specific for AD; and 3) to evaluate the cost-effectiveness of a case management program for AD patients and their caregivers.

## Methods/design

### Study design

UP-TECH is a multi-component, randomized, controlled trial, lasting 12 months, and enrolling dyads composed of an AD patient and their primary family caregiver. A total of 450 dyads, living in five different health districts of the Italian Marche region, will be randomized into three arms: 1) 150 dyads provided with systematic and comprehensive support of a trained social worker acting as case manager and receiving three nurse home visits (UP group); 2) 150 dyads provided with systematic and comprehensive support of a trained social worker acting as case manager, receiving an assistive technology intervention and three nurse home visits (UP-TECH group); and 3) 150 dyads receiving ‘light’ support in the form of a paper brochure and three nurse home visits (control group).

#### Eligibility criteria

AD patients are subjects with a pre-existing diagnosis of AD, in an intermediate stage, according to the 2011 criteria of the National Institute on Aging-Alzheimer's Association (NIA-AA) [32-34], with a mini-mental state examination (MMSE) score [35] between 10 and 20, living in the community, and assisted by at least one family caregiver.

Family caregivers are defined as those kin either living or not living with the patient (if not habitually living with the patient, then living in the same municipality), who care for and directly support the patient with the activities of daily living (ADL) and instrumental activities of daily living (IADL) for at least 1 hour per day within the last 6 months. Where there is more than one family caregiver, the primary caregiver, that is, the family member who spends more hours assisting the patient, will be included in the trial.

The exclusion criteria are: 1) lack of informed consent from the AD patient or caregiver. If the patient has been declared legally incompetent or has a support administrator appointed, informed consent will be requested from a family member or from a person appointed by a judge. In the case of natural incapacitation, verified by Alzheimer Evaluation Unit (AEU) doctors, consent for the patient will be requested from the primary caregiver; 2) presence of severe diseases associated with disability in ADLs, life expectancy less than 6 months in addition to AD, or unstable chronic conditions in both the AD patient and the family caregiver, as assessed by the AEU and other professionals in the health district; 3) intention of moving out of the health district within 12 months; 4) enrolled in another experimental trial; and 5) lack of a family caregiver or a caregiver less than 18 years old.

#### Outcomes of the study

The main outcomes of the study are: 1) caregiver burden, measured using the Caregiver Burden Inventory (CBI) multi-dimensional questionnaire, administered by a trained nurse. The CBI has five subscales: time-dependence burden, developmental burden, physical burden, social burden and emotional burden, and includes a set of 24 items evaluated using a 5-point Likert scale ranging from 0 (not at all) to 4 (very much), which are summed. A total score higher than 36 indicates a risk of stress, whereas scores near or slightly above 24 indicate a need to seek some form of respite care [36]. During the study, the Italian version of the CBI validated by Marvardi and colleagues will be used [37]; and 2) the actual number of days spent at home by the AD patient during the observation period, defined as days free from episodes of institutionalizations, inpatient hospitalizations and brief stays in an observation unit of an emergency room. This outcome will be treated as a continuous variable and will be calculated at each assessment (6 months, M6; 12 months, M12; and 24 months, M24) by subtracting from the total days of observation, the number of days of institutionalization in an assisted residence facility, care home and/or nursing home inpatient, hospitalizations, and brief stays in an observation unit of an emergency room.

In addition, the following outcomes will be evaluated at the end of the trial: 1) quality of life of the caregiver of the AD patient, measured using the Italian validated version of the quality of life questionnaire, SF-12 [38,39]; 2) anxiety and depression of caregivers, measured using the Italian validated version of the Hospital Anxiety and Depression Scale (HADS) [40,41]. HADS is a 14-item scale, seven of the items relate to anxiety and seven relate to depression, each item is a Likert scale scoring from 0 to 3, and this means that the overall score for either anxiety or depression will be between 0 and 21; and 3) abuse and mistreatment risk of AD patients, using the CASE instrument, an 8-item screening tool designed to assess caregivers for potential abuse. Answers are either given as ‘yes’ or ‘no’, and an answer of ‘yes’ for a question equals one point. A score of four or more is considered ‘abuse likely’, but a score of one can also mean that abuse is likely, depending on the question [42]. An Italian validation of this instrument is planned in a separate study at the end of the trial.

#### Sample size calculation

An estimation of the sample size was carried out by considering the two main outcomes separately.

The first principal outcome is caregiver burden. A previous Italian study estimated that the level of burden of AD patients' caregivers, as measured by the CBI [36], was equal to 32.5, with a standard deviation (SD) equal to 18 [37]. The planned sample size of 150 caregivers is sufficient to detect an effect that lowers the CBI score to 24, with a SD equal to 12 in the treatment group and a null effect in the control group. The statistical power was fixed at 80%, with a 0.05 significance level and a drop-out rate equal to 15%. A CBI score of 24 coincides with a ‘sentinel’ level beyond which it is suggested that caregivers need to receive additional support from the health care and social services.

The second principal outcome is actual days spent at home by the AD patient in the past year. The hypothesis that this outcome is the same in the treated and the untreated populations will be tested. The calculation was made considering a type 1 error (α error) of 0.05, using a one-tailed t-test and assuming a 10% difference between values. Regarding this as the smallest effect of clinical relevance, a sample of 150 patients per treatment group, with a drop-out rate equal to 15%, will be adequate to achieve an 80% probability of rejecting the null hypothesis.

#### Recruitment and screening

In the absence of a regional AD registry, lists of AD patients will be requested from AEUs of the five health districts (Pesaro, Ancona, Macerata, Fermo and San Benedetto del Tronto). The AEUs were established in Italy in 2000 with the aim of improving the care pathways for AD patients. AEUs are multidisciplinary teams, usually led by a neurologist or a geriatrician, with a double-fold task: 1) performing a diagnosis of AD according to the most recent clinical recommendations; and 2) prescribing cholinesterase inhibitors, memantine and atypical antipsychotics under the reimbursement scheme of the Italian health care system. Thus, the patients followed by the AEUs are those using the aforementioned drugs and receiving regular follow-ups by the physicians working in the units. These patients do not usually receive other care services, except from AEUs as consequences of AD. The AEUs will provide the lists of AD patients matching the inclusion criteria. Only prevalent patients will be extracted and eligible to participate in the study. Since local AD evaluation units do not share IT systems, a general list of patients will be created in order to randomly select subjects to be enrolled in the study. Inclusion and exclusion criteria will then be applied to those patients.

#### Randomization, allocation and blinding

A letter with an invitation to participate in the project will be sent to a list of potential participants, randomly extracted from the AEU registers. A total of 640 invitations will be sent, considering a response rate of 70% (a reasonably high rate, since the project has been endorsed by the Italian regional health system and several local patients' organizations). Families agreeing to be contacted will receive a home visit by a nurse. Both caregivers and patients will be requested to sign a full informed consent to participate in the study. The nurses who will make the home visits and administer the questionnaires will be blinded at their first visit.

**Figure 1:**
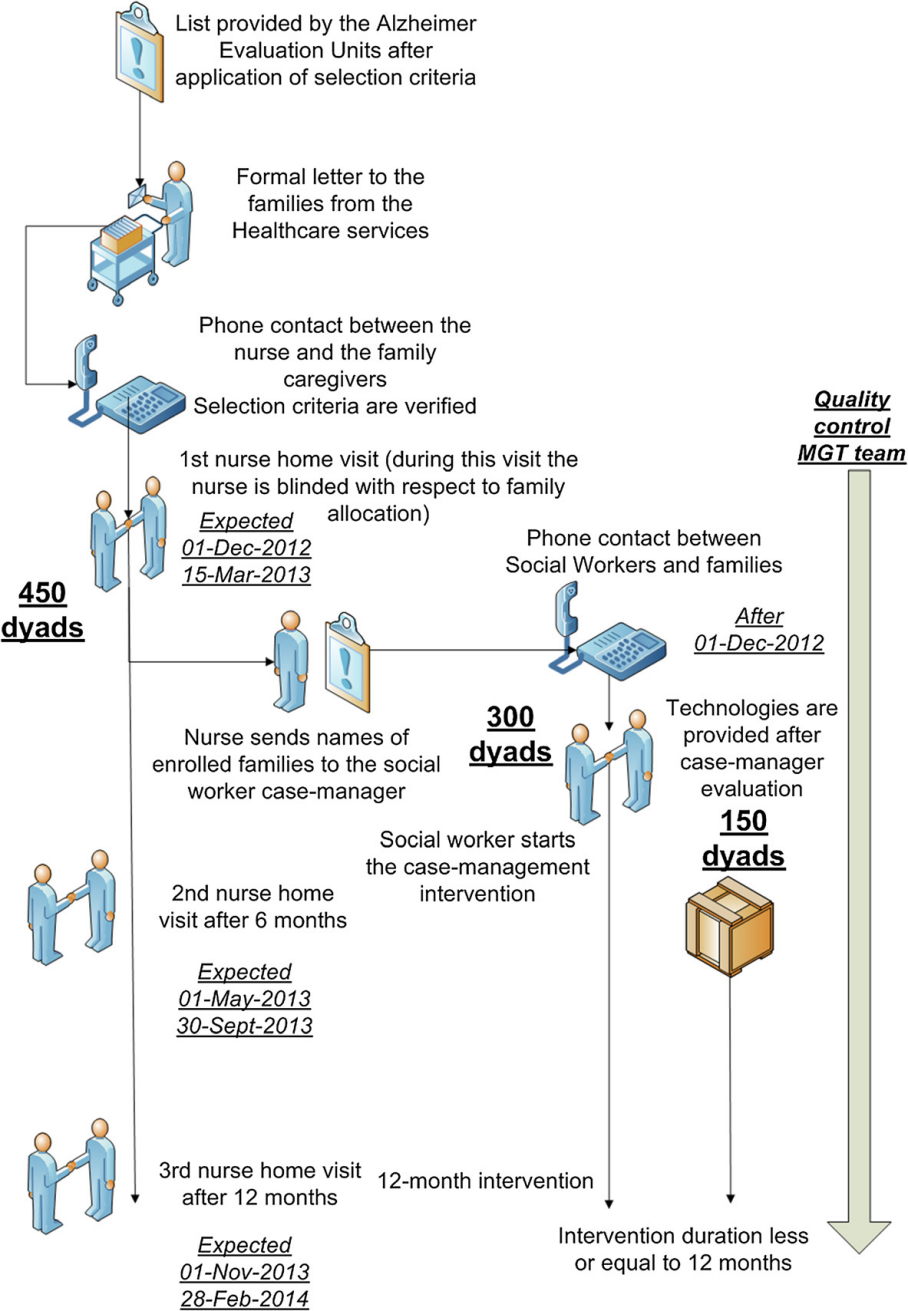
The recruitment process in the UP-TECH project.

The allocation sequence will be generated using the statistical software STATA (College Station, TX, USA). Dyads in blocks of 15 and consequently enrolled will be sent by a nurse coordinator in the five health districts directly to the project manager (CC). In each block, five dyads will be allocated to each arm. The randomization will be performed by the project manager, who will not conduct any assessment related to treatment outcomes or have any contact with line staff involved in the intervention, in order to preserve allocation concealment and prevent selection bias (Figure 1).

#### Interventions

### Case manager intervention

An *ad hoc* trained and hired case manager social worker will systematically and comprehensively support each patient-caregiver dyad in the UP and UP-TECH groups. The underlying concept of case management is inspired by the guidelines of the Case Management Society of America [43]. According to this definition, case management can be defined as ‘a collaborative process of assessment, planning, facilitation, care coordination, evaluation, and advocacy for options and services to meet an individual’s and family’s comprehensive health needs through communication and available resources to promote quality cost-effective outcomes’.

Before starting the trial, all case managers will attend an 8-day intensive and multidisciplinary course, which will address the following subjects: 1) clinical, psychological and social consequences of AD; 2) organization of health and social care services for AD patients; 3) fiscal and law benefits for AD patients; 4) counseling for caregivers; 5) methodology of multidisciplinary work; 6) bioethics; and 7) use of new technology for care. After the course, each case manager will meet on a monthly basis with a senior social worker, who will act as supervisor of the group in order to share experiences and receive advice on the most difficult cases.

The following support will be provided by a case manager: 1) at least three sessions of individual face-to-face counseling, focusing on topics such as housing arrangements, disease awareness and problem solving (following an initial meeting, two reinforcing sessions will be held at 4 and 8 months); 2) monthly follow-up telephone calls; 3) stress management training; 4) information about services/aid/certification/subsidies offered by the Italian national health service, municipal social services and local volunteer organizations (for example information on health care services, support connecting to general practitioners, health service units (medical specialists, hospital services) and social services); and 5) coordination of professionals working in different care settings, and between hospital and community doctors in the case of hospital discharge.

All these services will be addressed with both parties of the dyad and can be intensified when either the caregiver or the patient shows signs of anxiety, stress or poor health, which could also emerge during interviews. Each case manager will handle up to 30 dyads, out of a total of 300 dyads (150 in the UP group and 150 in the UP-TECH group). The placement of the case manager within the network of existing health care and social services in the community will be made following a thorough analysis of care processes in order to facilitate their integration.

### Technological device intervention

The project involves the testing of new technologies to support the patient and their caregiver, and the advice of a virtual information counter for technological aids and adapting to the home environment. The technologies to be employed are devices already widely used and marketed, are simple to use, and do not require significant technical expertise for installation and maintenance.

The technologies have been identified after a series of preliminary focus groups with caregivers and will include housing adaptations, such as luminous paths, home leaving sensors, sensors to detect night falls, gas and water leak sensors, and automatic lights. These devices will be assembled by an external contractor and linked to a single-board microcontroller, which will transmit alarm messages to the caregivers in case of need. The assembled devices will be assigned to subjects in the UP-TECH group after a joint evaluation of the home by the case manager and the engineer working in the virtual counter. Although these technologies can appear as low-tech in other industrialized countries, the degree of up-take of these devices in the houses of older Italians is almost non-existent.

**Figure 2:**
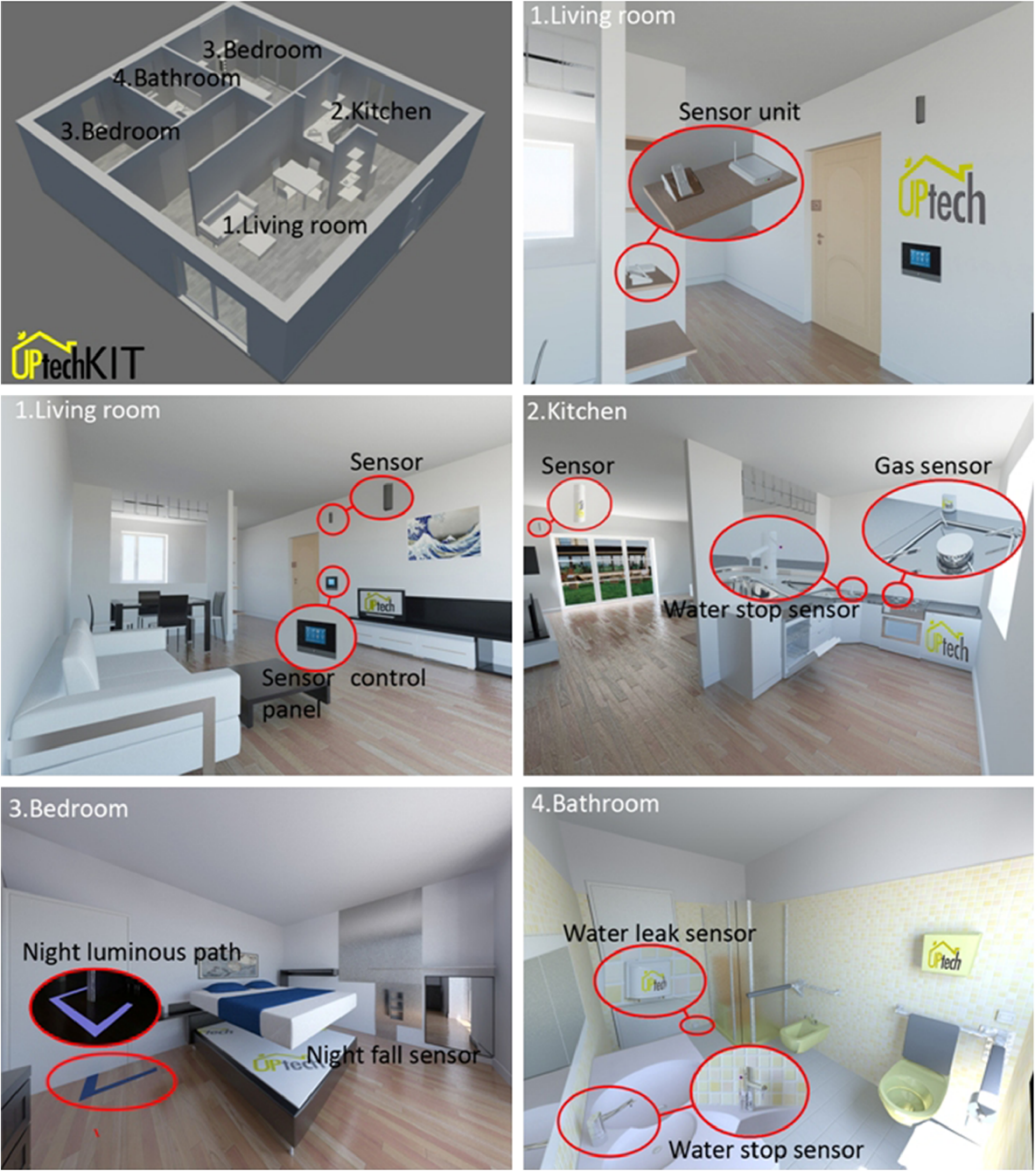
The design of the UP-TECH kit.

Assistive technology will be installed by the case manager with the support of expert technicians who will also train the caregivers about their use (Figure 2).

### Nurse home visits and light support

All dyads participating in the UP-TECH group will receive three home visits by a specifically trained nurse. Home visits will occur at enrollment (M0), 6 months (M6) and 12 months (M12). Each visit will occur with the following steps: 1) telephone contact between the nurse and the family caregiver; 2) collection of informed consent (only at M0) and administration of the UP-TECH questionnaire; and 3) brief counseling/training of the caregiver regarding practical aspects of patient assistance, such as daily management of drug treatment, ergonomics of the home environment, stress management and care burden.

The creation of an information package for the 150 family caregivers included in the control group, illustrating the range of social and health services available in the local community, is planned. The information package will be delivered to the caregiver during home visits by the nurse. These caregivers and their families will continue to receive services in accordance with the usual care procedure, which, as already mentioned, does not include any other specific service from the health and social care systems. Some patients might receive additional care services, but this does not occur within the framework of an integrated case management program, and will depend on the presence of other pathological and social conditions.

#### Assessments

All dyads will be interviewed using standardized tools. The interviewers will be the same nurses conducting the three home visits.

The first evaluation will also be the most in-depth. The UP-TECH ‘questionnaire 1’ is divided into three parts: the first focuses on AD patients (section A to C), the second part concerns caregivers (section D to H), while the final part measures the consumption of social and health care resources by the dyad (section I).

A nurse, trained to collect data, will administer a modified version of the interResident Assessment Instrument Contact Assessment (interRAI CA, Ann Arbor, MI, USA) to the AD patient, when possible, or to the caregiver, who will act as a proxy respondent [44]. The interRAI CA is a validated tool to support the decision-making process when managing the home and/or palliative care of the patient.

The instrument adapted from the interRAI CA will contain items designed to investigate the following areas: section A, general patient information: name, sex, date of birth, health district affiliation, housing conditions, surgery in the last 90 days and two main informal caregivers (questions about the benefits of the Italian legal system, Law 104/1992, were added); section B, screening information: the ability to make everyday decisions, ADLs, dyspnea, clinical status and perception of health status; and section C, clinical evaluation: changes in the ability to make everyday decisions, ability to understand other people, eyesight, reported indicators of depressed mood, behavioral problems, IADL difficulties, changes in ADL independence, disease diagnoses, falls, frequency of problems, pain, smoking, assessment of nutritional status, mode of eating, the highest stage of pressure ulcer, skin ulcer (not caused by pressure), other skin problems, traumatic injuries, treatments and services received, time since last hospitalization, and number of emergency room visits.

The family caregiver will be given a questionnaire consisting of five sections: section D, demographic and socio-economic status: age, sex, housing, income and social network support using the Multidimensional Scale of Perceived Social Support (MSPSS) [45]; section E, lifestyle: information regarding diet, smoking and alcohol consumption; section F, clinical evaluation: information on the perception of caregiver depression and anxiety (HADS) [40], the perception of well-being of the caregiver (quality of life questionnaire, SF-12) [38], and the presence of chronic diseases; section G. anthropometric measurements, including control of blood pressure and measurement of body mass index (BMI), via evaluation of height and weight; and section H, the caregiving relationship: caregiver burden measured with the CBI [36] and the risk of domestic abuse in the caregiving relationship [42].

In the last section of the questionnaire (section I, consumption of resources by the AD patient), the family caregiver will be asked for information regarding: resource consumption of the AD patient (for example drug therapy, visits to general practitioners and specialists, use of home nursing, and hospitalizations); use of treatment/care (for example nurse and formal caregiver) paid for by the patient/caregiver dyad, expressed in number of hours per week of treatment (or care); and the number of hours of work lost by the caregiver (hours per week) to provide assistance for the AD patient (productivity loss).

After the start of the study, at 6 months (M6) and 12 months (M12), respectively, a second and a third assessment will be conducted. Twelve months after the end of the trial (M24), follow-up telephone calls will be made to study participants in order to assess the impact of the interventions over the medium term.

#### Integration of collected data with Alzheimer Evaluation Units (AEUs)

The information collected through the questionnaire will be integrated with information already held by the AEUs. The following scales will be obtained for each patient: MMSE, Clinical Dementia Rating (CDR) and Neuropsychiatric Inventory (NPI). Information concerning hospitalizations and episodes of institutionalization in other residential care facilities will also be retrieved from regional hospital discharge and residential care databases.

#### Statistics

The first step of the analysis will be exploratory in nature. A descriptive analysis of the sample will be conducted using uni- and bi-variate statistical analyses, with the aim of verifying the comparability of the three study groups. Significant differences between exposures and outcomes will be compared using the chi-square test, Fisher’s exact test (in the case of categorical variables), t-test or analysis of variance (ANOVA) test (for comparisons of continuous variables between groups according to a normal or non-normal distribution). Additionally, to check possible selection bias, the characteristics of the subjects in the sample will be compared to those of non-respondents for available variables (for example age, gender, place of living and MMSE score).

The primary outcome, caregiver burden inventory, will be compared between the three groups using analysis of covariance (ANCOVA). The analyses will adjust for variables which are non-homogenously distributed in the three groups and control for other factors that the literature suggests are associated with the two outcomes (for example gender and age of caregivers, severity of dementia, and use of private care workers) [46,47].

The primary outcome, number of days spent at home by the AD patient during the observation period, will be similarly analyzed using ANCOVA. The events of hospitalizations and institutionalization will also be analyzed with the following: the distribution of events and event-free survival (days spent at home) will be described for the entire sample using Kaplan-Meier estimator modeling; Mantel-Cox log-rank test or generalized Gehan-Breslow-Wilcoxon test will be used to evaluate bivariate differences in survival distribution between identified subgroups; Cox proportional hazard modeling will be used to analyze the multivariate effects of identified risk factors and other determinants on time spent at home under the proportional hazards assumption; and adjusted hazards ratio (HR) and 95% confidence intervals (CI) will be calculated to test the direction and strength of the influence of individual factors on event-risk and event-free survival. The analyses of outcomes will be intention-to-treat (ITT).

#### Multiple comparison issues

The issue of multiple comparisons will be carefully considered when investigating further specific questions. This issue will be addressed using two strategies. First, statistical adjustments for multiple comparisons will be used (for example the Bonferroni correction for analyses with less than 10 comparisons or the Tukey's test in the other cases, both available on STATA) to strengthen the criterion for judging a result as significant and reduce the likelihood of false-positive results. Second, future secondary and subgroup analyses will always indicate all the subgroups and outcomes considered in the trials. We will differentiate exploratory, sensitivity and other *post hoc* analyses from pre-specified analyses of the primary and secondary outcomes discussed in the protocol, and report them in any future publications.

#### Health economics analysis

Cost, cost-effectiveness and cost-utility analyses will be performed using the perspective of the Italian national health service and municipalities. The cost-analysis will be stratified according to allocation group (UP, UP-TECH and control groups) and will include direct costs in terms of: 1) time spent by each social worker, for each patient/caregiver dyad; 2) time spent by other professionals with each patient; 3) time spent training social workers to perform the case management intervention; 4) time and other costs of social workers and nurses for travel; 5) use of drugs for dementia and related costs; 6) use of other drugs and related costs; 7) use of health care and social services; 8) cost of other consumables used for the interventions; and 9) cost of technological devices.

In a separate analysis we will also consider the social costs of AD, defined as the sum of costs incurred providing formal treatment and patient care plus the cost of informal care and the loss of productivity of caregivers. The assistance provided by family caregivers will also be assessed by valuing it equal to the salary of an unskilled care assistant.

The availability of cost data, together with data collection on health care outcomes, such as time to institu-tionalization of the patient and the stress level/quality of life of the caregiver, will allow a cost-effectiveness and cost-utility analysis. We will consider the control group (light care intervention) as the reference group for incremental analysis.

As outcomes measures for economic analysis we will use: 1) change in caregiver burden over a 12-month period; 2) the number of institutionalizations; 3) hospitalizations; and 4) deaths. Since both the costs and the effects of the interventions will occur over a period of 1 year, it will not be necessary to adjust for the time delay and to discount the measures. Four incremental cost-effectiveness ratios (ICERs) will then be calculated by taking the difference in costs divided by the difference in the four outcomes between the groups. The ICERs will represent the additional costs of gaining an additional unit of the above outcome measures through the intervention owing to the case management intervention and the case management plus the technological intervention.

In a following step, probabilistic sensitivity analyses (PSA) will also be performed on the three models using the software, TreeAge Pro 2009 (Williamstown, MA, USA). Results from the PSA will also be presented as an acceptability curve, graphically illustrating the probability of the intervention being cost-effective over a range of willingness to pay values.

A cost-utility analysis will estimate the ratio between the cost of an intervention and the benefit it produces in terms of caregivers' quality-adjusted life years (QALYs). Utility will be measured using the SF-12 administered during the nurse visits [48]. The PSA of this model will follow a similar procedure to that of the cost-effectiveness analysis.

## Discussion

The implementation of the UP-TECH project responds to the increasing need to cope with the consequences of the AD epidemic. It is in line with present policy and research priorities at both Italian and European levels [49,50].

Findings in this project are intended to support both health care and social service professionals and policy-makers in the enactment of future programs, addressing the increasing needs of AD patients and their caregivers. The rationale behind the project is that, despite the growing literature in this area, several controversies still need to be resolved and effective options need to be laid out in order to both avoid the institutionalization of AD patients and the burn-out of informal caregivers [11].

The project gathers the existing knowledge and related strategies in the area of dementia care, that is, the use of case management programs [8,15,16,26], new technologies [8,20,26], preventive home visits [51] and efforts toward integration of existing services [27-30], and tries to integrate them in an ambitious holistic design.

Possible limitations of the design should be mentioned. As the caregiver-patient dyads are allocated after the full consent, blinding is not required during the first home visit; however, blinding cannot be guaranteed during the second and third visits, or during the follow-up telephone call. Since the nurses will engage with patients and caregivers in long conversations, it is likely that they will be told about the social worker intervention or they will notice the technological toolkit installed in the home. In order to minimize the risk of performance and ascertainment bias, we will provide an *ad hoc* training course to nurse assessors, and we will keep as separated as possible the professionals intervening on the patients and caregivers (the social workers) from those assessing the outcomes (the nurses). In addition, we preserved the solidity of the study by choosing as one of the two primary outcomes, the number of days free from hospitalizations and other institutionalization episodes, a measure which is not likely to be influenced by an eventual un-blinding of the assessors.

Regarding the use of new technologies, the UP-TECH project does not entail the use of highly complex and innovative devices, but aims to test the effectiveness of simple marketed technological devices combined with case management and user-centered strategies.

Another key aspect of this investigation is the combination of research together with the implementation of a patient-based IT system in the Marche region of Italy. This will consolidate information, allowing community and hospital doctors, as well as social workers and other professionals, to coordinate health care and social services for AD patients and their caregivers. This IT-based integration of information is therefore expected to increase the efficiency and efficacy of deploying health care and social services, which is especially important in an environment of increasing demand and decreasing resources.

Finally, the costs and the cost-effectiveness of different interventions will also be evaluated through detailed analyses of formal and informal resource consumption during the study. The evaluation will adopt the perspectives of both the Italian national health service and of Italian society. This will allow testing of the hypothesis that the cost-effectiveness of the programs addressing AD patients in the community also remains even when considering hidden costs of care, such as caregiving burden and loss of productivity. This is expected to provide sound and definitive evidence on the costs and cost-effectiveness of programs targeting AD patients in the community, thus allowing a comparison of institutional and community-based care solutions, and address future reforms of the local and national welfare system.

### Trial status

The trial will start recruiting on 1 December 2012 and is scheduled to continue until 31 March 2014.

## Abbreviations

CBI: Caregiver Burden Inventory; ADL: Activities of daily living; AEU: Alzheimer Evaluation Unit; ANCOVA: analysis of covariance; ANOVA: Analysis of variance; BMI: Body mass index; CDR: Clinical Dementia Rating; CI: Confidence intervals; HADS: Hospital Anxiety and Depression Scale; HR: Hazards ratio; IADL: Instrumental activities of daily living; ICER: Incremental cost-effectiveness ratios; INRCA: Italian National Research Center on Aging; ITT: Intention-to-treat; interRAI CA: InterResident Assessment Instrument Contact Assessment; Istat: Italian National Institute of Statistics; IT: Information technology; MMSE: Mini-mental state examination; MSPSS: Multidimensional Scale of Perceived Social Support; NIA-AA: National Institute on Aging-Alzheimer's Association; NPI: Neuropsychiatric Inventory; PSA: Probabilistic sensitivity analyses; QALY: Quality-adjusted life year; SD: Standard deviation

## Competing interests

The authors declare that they have no competing interests.

## Authors’ contributions

CC: study concept; project manager of the study; preparation of study protocol; drafting of manuscript. FM: principal investigator of the study; preparation of study protocol; critical review of manuscript. JMR: preparation of study protocol; critical review of manuscript; English language editing. AC: preparation of study protocol; critical review of manuscript. OS: preparation of study protocol; critical review of manuscript. LS: preparation of study protocol; critical review of manuscript. FL: preparation of study protocol; critical review of manuscript. All authors read and approved the final manuscript.
